# Immune Cells and Intracerebral Hemorrhage: A Causal Investigation Through Mendelian Randomization

**DOI:** 10.1002/brb3.70263

**Published:** 2025-01-10

**Authors:** Liumei Mo, Wei Pan, Wenjing Cao, Kui Wang, Li'an Huang

**Affiliations:** ^1^ Department of Neurology The First Affiliated Hospital Jinan University Guangzhou Guangdong China; ^2^ Department of Cardiology Foshan Women and Children Hospital Foshan Guangdong China; ^3^ Department of Geriatrics Foshan Women and Children Hospital Foshan Guangdong China; ^4^ The First Clinical Medical College Shandong University Jinan Shandong China

**Keywords:** genetic variants, genome‐wide association study, immune cells, intracerebral hemorrhage, MR analyses

## Abstract

**Background:**

The involvement of immune cells in the pathophysiology of intracerebral hemorrhage (ICH) is becoming increasingly recognized, yet their specific causal contributions remain uncertain. The objective of this research is to uncover the potential causal interactions between diverse immune cells and ICH using Mendelian randomization (MR) analysis.

**Methods:**

Genetic variants associated with 731 immune cell traits were sourced from a comprehensive genome‐wide association study (GWAS) involving 3757 participants. Summary statistics data for ICH were acquired from FinnGen, comprising 4056 ICH cases and 371,717 controls. The principal analytical tool utilized in our study was the inverse‐variance weighted (IVW) method, incorporated as a key component of a two‐sample MR approach. To mitigate potential biases and verify the stability of the conclusions drawn from the primary analytical methods, a series of sensitivity analyses were performed.

**Results:**

MR analysis elucidated 33 immune cell traits with causal associations, comprising B cells (eight traits), conventional dendritic cells (cDC, two traits), maturation stages of T cells (two traits), monocytes (two traits), myeloid cells (five traits), TBNK cells (six traits), and regulatory T cells (Treg, eight traits). DP (CD4+CD8+) %T cell (OR = 0.83, CI = 0.72–0.96, *p* = 0.013) exhibited the strongest protective effect. In contrast, transitional AC (OR = 1.09, CI = 1.02–1.16, *p* = 0.006) and IgD− CD27− %lymphocyte (OR = 1.08, CI = 1.00–1.17, *p* = 0.045) showed a higher tendency to increase the ICH risk. The sensitivity analyses validated the robustness and consistency of these results.

**Conclusion:**

Our research provides robust evidence substantiating the causal relationship between specific immunophenotypes and ICH risk. The identification of these findings significantly enhances our understanding of the pathogenic mechanisms underlying ICH, particularly pertaining to the immune system. This breakthrough paves the way for innovative clinical and pharmaceutical research opportunities, potentially promoting the development of targeted therapies and enhanced strategies for managing and preventing ICH.

## Graphical abstract

This study used the Mendelian randomization to identify causal associations between specific immune cell traits and intracerebral hemorrhage (ICH) risk, highlighting potential immune cell phenotypes as novel targets for future therapeutic interventions in ICH.

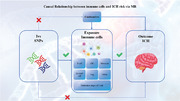

**Abbreviations**
BBB: blood–brain barrier
CI: confidence interval
CNS: central nervous system
GWAS: genome‐wide association study
ICH: intracerebral hemorrhage
IV: instrumental variable
IVW: inverse‐variance weighted
MR: Mendelian randomization
OR: odds ratio
SNP: single nucleotide polymorphism

## 1 Introduction

Intracerebral hemorrhage (ICH), often referred to as hemorrhagic stroke, is a condition distinguished by the spontaneous bleeding within the brain parenchyma, typically resulting from ruptured blood vessels. ICH accounts for approximately 10%–15% of all stroke cases and has significantly higher mortality and morbidity rates compared to ischemic stroke. Statistics reveal that the early mortality rate for ICH can reach as high as 40%, and those who survive often experience severe long‐term neurological deficits and a reduction in quality of life (Magid‐Bernstein et al. 2022). As a substantial contributor to global medical burden, the incidence of ICH escalates with age and is particularly prevalent in individuals with hypertension, the most significant modifiable risk factor. Beyond hypertension, other factors such as cerebral amyloid angiopathy (CAA), cerebral arteriovenous malformation (AVM), coagulation disorders, and cerebral aneurysms may also contribute to ICH. Given its high‐level morbidity and mortality, there is an urgent need for effective therapeutic interventions and a deeper understanding of its pathophysiology. Early intervention and preventive measures are crucial in mitigating the impact of ICH on individuals and public health as a whole (GBD 2019 Stroke Collaborators 2021).

Recent research has increasingly demonstrated the crucial contribution of the immune system to the onset and advancement of ICH (An, Kim, and Yoon 2017). Studies have shown that following the onset of hemorrhage, blood constituents surge into the brain parenchyma, which in turn triggers a robust inflammatory response. Immune cells like macrophages, neutrophils, and T cells are rapidly recruited to the hemorrhagic region, where they release various inflammatory substances and cytokines (K. Shi et al. 2019). These immune responses are double‐edged: they can facilitate the clearance of hematoma and promote tissue repair, but they can also exacerbate secondary brain injury, resulting in worse clinical outcomes. For instance, neutrophils have the potential to worsen secondary brain injury via the release of reactive oxygen species (ROS), proteolytic enzymes, and pro‐inflammatory mediators. Macrophages exhibit plasticity in their polarization states (M1 and M2 phenotypes), playing different roles at different stages of the inflammatory response (Murray and Wynn 2011). T cells, via their regulatory mechanisms, are capable of modulating the local inflammatory environment and influencing the overall trajectory of brain recovery post‐ICH (S. Chen et al. 2013; Yang et al. 2014). Given the aforementioned dynamics, acquiring a thorough comprehension of the specific mechanisms by which immune cells influence the outcomes of ICH is paramount. Such insights not only enhance our understanding of ICH pathophysiology but also facilitate the identification of pioneering therapeutic targets that regulate the immune response, ultimately improving patient prognosis.

Despite the valuable insights gained from observational studies on the involvement of immune cells in ICH, the inherent limitations of these studies and the complexity of the underlying pathological mechanisms have hindered a complete understanding of the specific functions and impacts of these cells. Prior observational studies have suggested a potential link between immune cell activity and ICH, but they are susceptible to confounding bias and reverse causality (Bowden and Holmes 2019). To tackle these challenges, our study employs Mendelian randomization (MR), a rigorous methodology that uses genetic variations as instrumental variables (IVs) to elucidate the causal involvement of immune cells in ICH pathogenesis. By scrutinizing genetic information from openly accessible online genome‐wide association studies (GWASs), MR offers enhanced precision and reliability by minimizing measurement errors and confounding variables (Orrù et al. 2020; Hu et al. 2024; Cao et al. 2024). By minimizing confounding and establishing causal directions, MR can determine whether preexisting changes in immune cell levels contribute to ICH pathogenesis or whether observed immune responses are a consequence of the hemorrhagic event. This strategy is of great importance for elucidating the intricate roles of diverse immune cell phenotypes in ICH and could facilitate the expeditious advancement of targeted therapeutic approaches.

## 2 Materials and Method

### 2.1 Study Design

**FIGURE 1:**
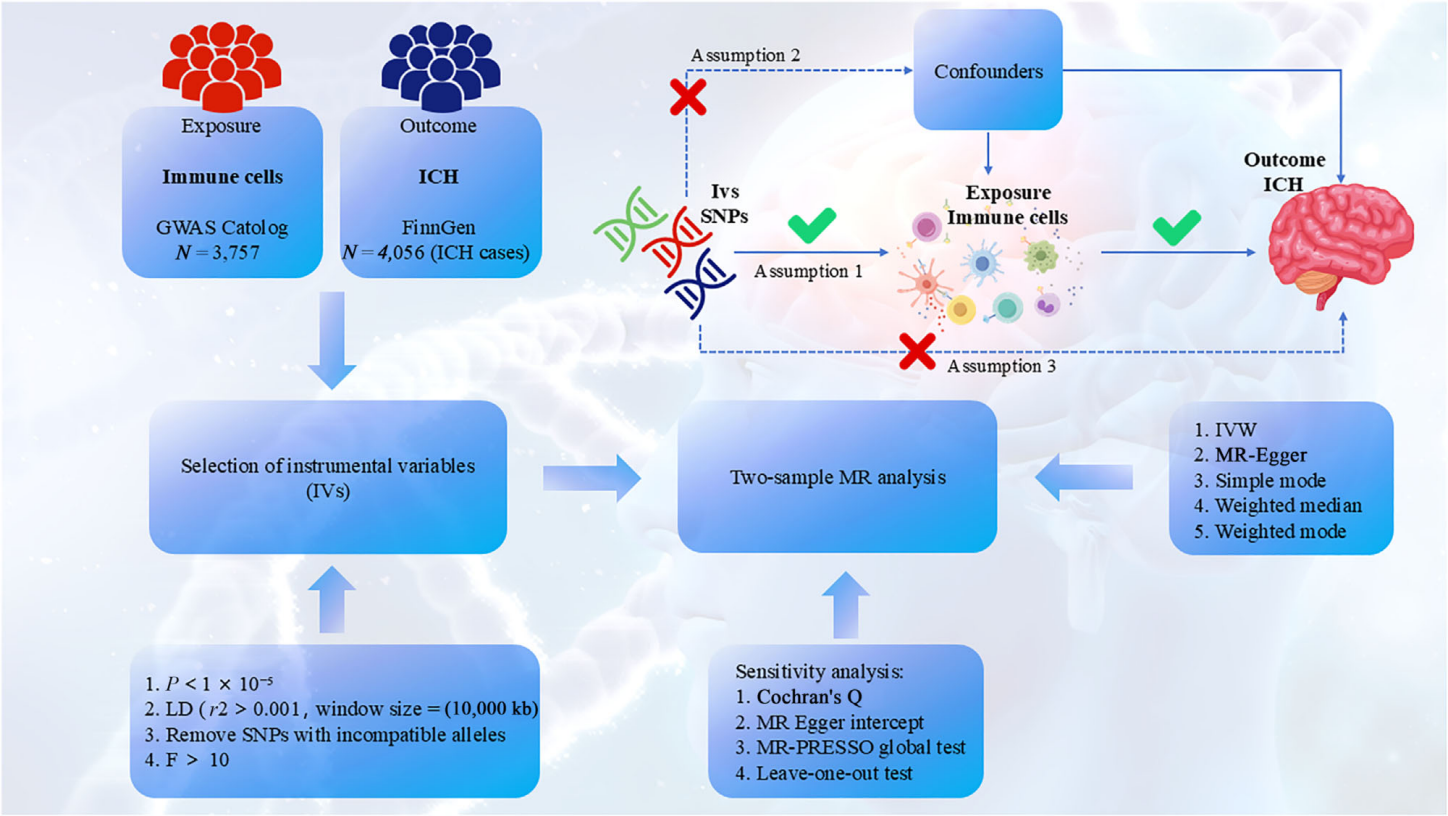
An overview of the study design.

Within this research, a comprehensive two‐sample MR analysis was conducted, wherein 731 immune cell traits served as the exposure variable, while ICH was investigated as the outcome to illuminate their potential causal relationship. In this method, genetic variants, specifically selected single nucleotide polymorphisms (SNPs), are used as IVs to effectively represent the exposure variable. For IVs to be considered valid in causal inference, they must simultaneously satisfy three critical assumptions: (1) IVs must exhibit significant correlation with the exposure (immune cells), (2) IVs must not exert any influence on confounders linking the exposure (immune cells) and the outcome (ICH), and (3) IVs must not impact the outcome via alternative pathways (Figure 1) (Davey Smith and Hemani 2014).

### 2.2 Data Sources

The GWAS dataset for ICH was sourced from the FinnGen consortium R10. The dataset comprised information from 375,773 Finnish individuals, consisting of 4056 ICH cases and 371,717 controls. Researchers interested in accessing this dataset can do so through the following website: https://r10.finngen.fi/pheno/I9_ICH.

The GWAS dataset for immune cell traits was publicly accessible through the GWAS Catalog, covering a comprehensive range of data from accession numbers GCST0001391 to GCST0002121 (Orrù et al. 2020). The initial GWAS dataset on immune traits was conducted utilizing information from 3757 individuals of European descent, without any overlapping cohorts (Sidore et al. 2015). The dataset comprised 731 distinct immunophenotypes, which were further categorized into four distinct groups: 118 absolute cell counts (AC), 389 median fluorescence intensities (MFI), 32 morphological parameters (MP), and 192 relative cell counts (RC). Specifically, the AC, MFI, and RC features included a diverse array of immune cells such as conventional dendritic cells (cDC), B cells, maturation stages of T cells, myeloid cells, monocytes, TBNK (T cells, B cells, natural killer [NK] cells), and regulatory T cells (Treg). Correspondingly, the MP features pertained to cDC and TBNK panels.

### 2.3 Selection of IVs

SNPs strongly associated with immune cell traits were identified using a stringent locus‐wide significance threshold of *p* < 1 × 10⁻⁵. Furthermore, datasets were harmonized by excluding variants in linkage disequilibrium (LD) (*r* ^2^ < 0.001 within 10,000 kb clumping distance). This process ensured that the selected IVs were independent and did not introduce bias into the analysis. The exposure and outcome effect estimates were standardized, while SNPs with incompatible alleles or palindromic sequences were systematically removed to ensure data quality. To guarantee the strength of the IVs and to counteract the bias of weak instruments, we derived the *F*‐statistic. An *F*‐statistic greater than 10 was commonly regarded as indicating strong IVs (Table S1).

### 2.4 Statistical Analyses

Utilizing MR analysis, this study endeavored to assess the causal correlation between 731 immunophenotypes and ICH. For features with multiple IVs, five widely accepted MR methods were implemented: inverse‐variance weighted (IVW) test (Burgess, Small, and Thompson 2017), MR‐Egger (Yavorska and Burgess 2017), simple mode (Hartwig, Davey Smith, and Bowden 2017), weighted median (Bowden et al. 2016), and weighted mode. These methods were chosen to address different assumptions about pleiotropy, with IVW being the primary method due to its efficiency under the assumption of no horizontal pleiotropy.

Subsequent to the identification of notable associations, sensitivity analyses were initiated to uncover potential heterogeneity and pleiotropy. Notably, the Cochran's *Q*‐test was employed to scrutinize heterogeneity, whereas the MR‐Egger intercept test and the MR‐PRESSO global test were utilized to evaluate horizontal pleiotropy (Verbanck et al. 2018; K. Wang et al. 2023). The absence of heterogeneity or pleiotropy was indicated by a *p* value exceeding 0.05. Conducting leave‐one‐out analyses enabled the assessment of whether any single SNP influences the estimation of causality. The funnel plots were utilized to illustrate the heterogeneity of SNPs, particularly assessing the symmetry of points on both sides of the IVW line. The scatter plots were utilized to visualize the findings of MR analysis investigating the relationship between the exposure factor (immune cells) and the outcome (ICH). Each data point on the plot corresponded to an instrumental variable SNP, with the actual response depicted by the 95% confidence interval (CI). The horizontal axis quantified the SNP's influence on the exposure (immune cells), while the vertical axis quantified the SNP's impact on the outcome (ICH). The ratio of these two effects elucidated the influence of the exposure (immune cells) on the outcome (ICH), with a slope less than zero indicating a beneficial effect and a slope greater than zero suggesting an adverse effect. Various algorithms employed in the analysis were represented by different colored lines. These analyses were executed with the R 4.2.3 software.

## 3 Results

### 3.1 Correlation Between Immune Cells and ICH

**FIGURE 2:**
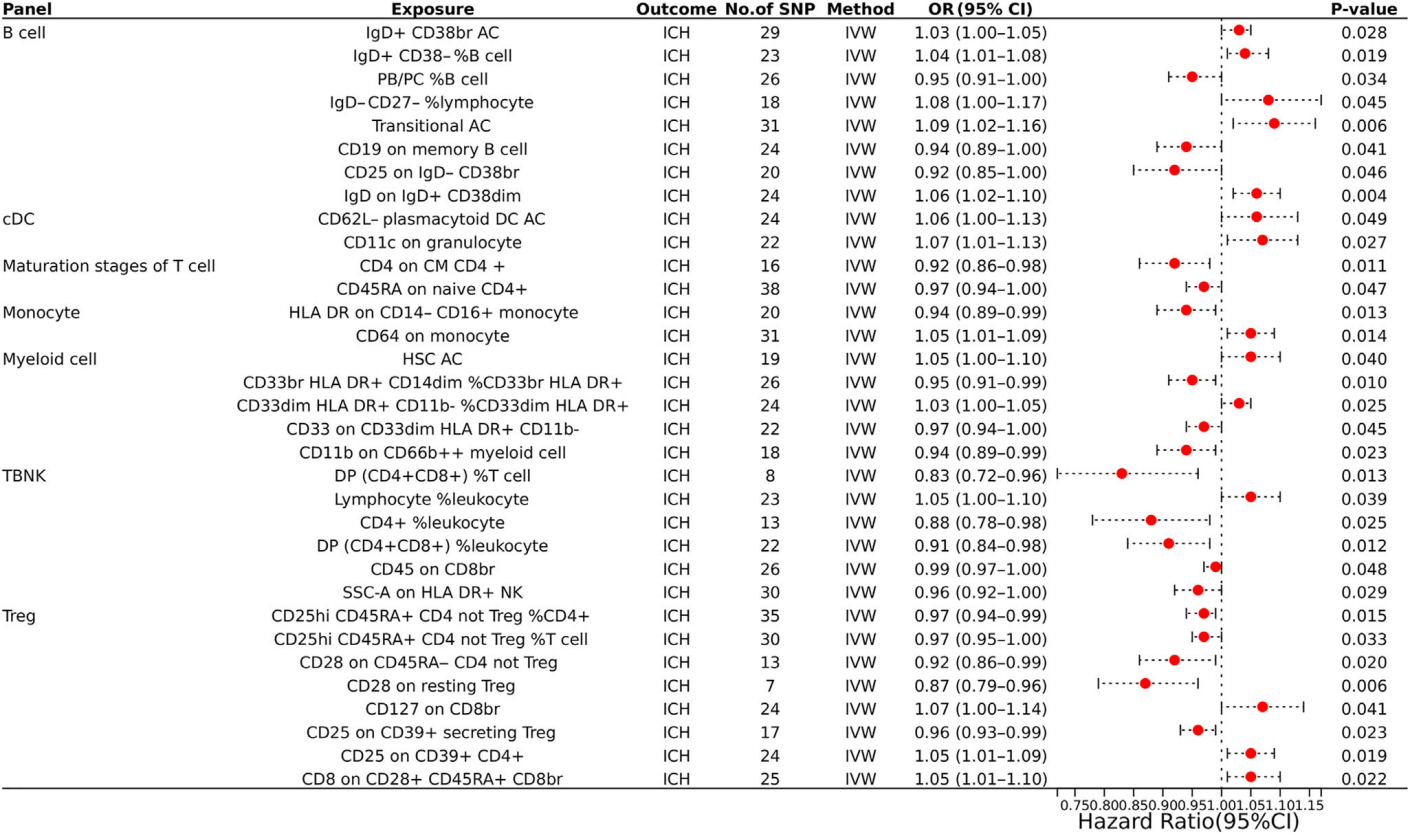
Forest plot of the MR analysis results using the IVW method.

After performing preliminary analyses to investigate the association between genetically predicted immune cell characteristics and ICH risk, primarily using the IVW method, we identified potential causal relationships in seven immune cell panels: eight within the B cell, two within the cDC, two within the maturation stages of T cell, two within the monocyte, five within the myeloid cell, eight within the Treg, and six within the TBNK, totaling 33 distinct immune cell traits. Among them, 19 immunophenotypes exhibited protective effects against ICH, whereas 14 immunological cell subtypes were associated with an increased incidence. For instance, DP (CD4+CD8+) %T cell (odds ratio [OR] = 0.83, CI = 0.72–0.96, *p* = 0.013) was associated with the most significant protective effect against ICH. In contrast, transitional AC (OR = 1.09, CI = 1.02–1.16, *p* = 0.006) and IgD− CD27− %lymphocyte (OR = 1.08, CI = 1.00–1.17, *p* = 0.045) tended to have a higher risk of ICH. Table 1 provides a summary of the key findings from the IVW analysis, and Figure 2 presents a forest plot depicting the associations of 33 immune cell characteristics with ICH.

**TABLE 1:** Summary results of MR.

Panel	Immune cells	No. of SNP	Methods	Beta	OR (95%CI)	*p* value	Heterogeneity		Horizontal Pleiotropy	
							Cochran's *Q*	*p* value	Egger intercept *p* value	Global *p* value
B cell	IgD+ CD38br AC	29	IVW	0.028	1.03 (1.00–1.05)	0.028	25.66	0.592	0.871	0.716
	IgD+ CD38− %B cell	23	IVW	0.041	1.04 (1.01–1.08)	0.019	28.65	0.155	0.954	0.289
	PB/PC %B cell	26	IVW	−0.046	0.95 (0.91–1.00)	0.034	19.53	0.771	0.775	0.789
	IgD− CD27− %lymphocyte	18	IVW	0.078	1.08 (1.00–1.17)	0.045	23.97	0.12	0.674	0.148
	Transitional AC	31	IVW	0.084	1.09 (1.02–1.16)	0.006	30.34	0.448	0.369	0.517
	CD19 on memory B cell	24	IVW	−0.06	0.94 (0.89–1.00)	0.041	29.27	0.171	0.095	0.173
	CD25 on IgD− CD38br	20	IVW	−0.083	0.92 (0.85–1.00)	0.046	20.91	0.342	0.279	0.361
	IgD on IgD+ CD38dim	24	IVW	0.058	1.06 (1.02–1.10)	0.004	12.89	0.954	0.497	0.928
cDC	CD62L− plasmacytoid DC AC	24	IVW	0.059	1.06 (1.00–1.13)	0.049	30.03	0.148	0.934	0.171
	CD11c on granulocyte	22	IVW	0.065	1.07 (1.01–1.13)	0.027	16.64	0.733	0.76	0.739
Maturation stages of T cell	CD4 on CM CD4+	16	IVW	−0.083	0.92 (0.86–0.98)	0.011	19.75	0.182	0.643	0.215
	CD45RA on naive CD4+	38	IVW	−0.034	0.97 (0.94–1.00)	0.047	35.38	0.545	0.81	0.556
Monocyte	HLA DR on CD14− CD16+ monocyte	20	IVW	−0.063	0.94 (0.89–0.99)	0.013	21.2	0.326	0.461	0.371
	CD64 on monocyte	31	IVW	0.047	1.05 (1.01–1.09)	0.014	28.19	0.561	0.631	0.538
Myeloid cell	HSC AC	19	IVW	0.05	1.05 (1.00–1.10)	0.04	17.58	0.483	0.172	0.538
	CD33br HLA DR+ CD14dim %CD33br HLA DR+	26	IVW	−0.055	0.95 (0.91–0.99)	0.01	31.66	0.168	0.607	0.211
	CD33dim HLA DR+ CD11b− %CD33dim HLA DR+	24	IVW	0.028	1.03 (1.00–1.05)	0.025	23.73	0.419	0.617	0.386
	CD33 on CD33dim HLA DR+ CD11b−	22	IVW	−0.034	0.97 (0.94–1.00)	0.045	13.73	0.881	0.422	0.786
	CD11b on CD66b++ myeloid cell	18	IVW	−0.064	0.94 (0.89–0.99)	0.023	14.75	0.614	0.912	0.636
TBNK	DP (CD4+CD8+) %T cell	8	IVW	−0.185	0.83 (0.72–0.96)	0.013	3.6	0.825	0.196	0.786
	Lymphocyte %leukocyte	23	IVW	0.048	1.05 (1.00–1.10)	0.039	32	0.077	0.186	0.131
	CD4+ %leukocyte	13	IVW	−0.13	0.88 (0.78–0.98)	0.025	10.16	0.602	0.727	0.631
	DP (CD4+CD8+) %leukocyte	22	IVW	−0.096	0.91 (0.84–0.98)	0.012	22.44	0.375	0.165	0.388
	CD45 on CD8br	26	IVW	−0.014	0.99 (0.97–1.00)	0.048	32.84	0.135	0.792	0.101
	SSC‐A on HLA DR+ NK	30	IVW	−0.042	0.96 (0.92–1.00)	0.029	25.91	0.63	0.244	0.691
Treg	CD25hi CD45RA+ CD4 not Treg %CD4+	35	IVW	−0.033	0.97 (0.94–0.99)	0.015	42.42	0.152	0.29	0.203
	CD25hi CD45RA+ CD4 not Treg %T cell	30	IVW	−0.029	0.97 (0.95–1.00)	0.033	29.99	0.415	0.683	0.44
	CD28 on CD45RA− CD4 not Treg	13	IVW	−0.082	0.92 (0.86–0.99)	0.02	11.17	0.515	0.308	0.584
	CD28 on resting Treg	7	IVW	−0.139	0.87 (0.79–0.96)	0.006	6.35	0.385	0.71	0.531
	CD127 on CD8br	24	IVW	0.068	1.07 (1.00–1.14)	0.041	21.2	0.569	0.353	0.621
	CD25 on CD39+ secreting Treg	17	IVW	−0.041	0.96 (0.93–0.99)	0.023	10.26	0.853	0.99	0.83
	CD25 on CD39+ CD4+	24	IVW	0.048	1.05 (1.01–1.09)	0.019	25.53	0.324	0.748	0.426
	CD8 on CD28+ CD45RA+ CD8br	25	IVW	0.053	1.05 (1.01–1.10)	0.022	26.53	0.327	0.895	0.353

### 3.2 Sensitivity Analysis

Sensitivity analysis was ultimately carried out for the results, showing no significant heterogeneity among the IVs based on Cochran's IVW *Q*‐test (*p* > 0.05, Table 1). Furthermore, neither the MR‐Egger intercept test nor the MR‐PRESSO global test showed evidence of horizontal pleiotropy, as indicated by *p* values greater than 0.05 (Table 1). The visualization of sensitivity analysis was demonstrated in Figure S1. Similarly, leave‐one‐out sensitivity analyses indicated that no single SNP exerted a notable influence on the causal association, as shown in Figure S2.

## 4 Discussion

In this study, we used an MR approach to explore causal relationships between 731 immune cell phenotypes and ICH, utilizing data from publicly available GWAS datasets. To our knowledge, no prior MR analysis has examined such a wide range of immune phenotypes in relation to ICH. Our findings identified 33 immune cell phenotypes with distinct effects on ICH risk, including 19 associated with protective effects and 14 linked to an increased risk of ICH.

The immune system plays an indispensable role throughout the pathophysiology of ICH. The process of neurological injury after ICH involves both primary and secondary mechanisms: primary injury includes hematoma expansion, cerebral edema, and elevated intracranial pressure, while secondary injury arises from oxidative stress, cytotoxicity, and inflammatory responses (Zhu, Wang, and Yu 2019; Z. Li et al. 2022). The immune response significantly influences ICH progression, severity, and patient outcomes (S. X. Shi, Vodovoz, et al. 2022). Significant research in recent years has focused on the importance and function of microglia/macrophages, astrocytes, and neutrophils in ICH (Zhang et al. 2024). However, the impact of T‐cell activity in the acute phase of ICH has also garnered considerable attention.

Although the exact mechanisms and extent of T‐cell involvement in ICH remain unclear, clinical and experimental studies have demonstrated their role in secondary injury and pathophysiology (S. X. Shi, Vodovoz, et al. 2022). Histological evidence from human and animal studies shows that CD4+ T cells dominate the infiltrating lymphocytes in ICH, while CD8+ T cell infiltration is less prominent (S. X. Shi et al. 2023). γδ T cells, activated by macrophage‐derived IL‐23, produce IL‐17, which may initially aid in clearing debris but, if prolonged, can worsen brain tissue damage and blood–brain barrier (BBB) disruption, exacerbating injury (Zhong et al. 2016). In contrast, Treg, differentiated from naive CD4+ T cells, exhibit protective effects (H.‐Y. Wang et al. 2022). In animal models of ICH, Treg (FoxP3+CD25+CD4+) have shown anti‐inflammatory properties, including inhibition of microglial activation, enhancement of BBB integrity, reduction of cerebral edema, and decreased cell death (Mao et al. 2017). Additionally, Treg cells modulate microglial and lymphocyte activation and reduce the production of proinflammatory cytokines, offering therapeutic potential (Liesz et al. 2009). Research has shown that while certain T cells can exacerbate damage through proinflammatory actions, they also help regulate inflammation and promote tissue repair (S. X. Shi, Vodovoz, et al. 2022). However, the exact mechanisms by which T cells contribute to ICH remain unclear, and the roles of different T cell subtypes in hemorrhagic brain injury are still poorly understood. Our study is the first to use MR to explore causal relationships between T cell subtypes and ICH. For example, we analyzed subtypes like CD28 on resting Treg, CD8 on CD28+CD45RA+CD8br, and CD127 on CD8br. Notably, most T cell immunophenotypes showed protective effects against ICH, with only a few linked to increased risk. These findings provide a foundation for further research into the specific roles of T‐cell subtypes in ICH pathogenesis and their potential as therapeutic targets.

The role of B cells in acute central nervous system (CNS) injury is complex, with diverse functions depending on their subtype, timing of infiltration, and pathological context (Zera and Buckwalter 2020). In ischemic stroke, B lymphocytes inhibit the recruitment and activation of other immune cells, thereby reducing infarct size and attenuating neurological deficits. Regulatory B cells further contribute to immunosuppression via production of IL‐10, IL‐35, and TGF‐β, aiding in the repair of ischemic brain tissue (Selvaraj et al. 2016). An observational study documented a decline in circulating B cells during the acute phase of stroke, correlating with the severity of neurological deficits (Daglas et al. 2019). These findings imply that B cells may have a neuroprotective function in disease development by reducing and modulating the inflammatory response. However, in the chronic phase of ischemic stroke, activated B lymphocytes infiltrate infarcted areas, producing antibodies such as IgA and IgG that contribute to delayed cognitive impairment (Maheshwari, Dwyer, and Sîrbulescu 2023). Pharmacological depletion of B cells using drugs like rituximab or genetic modifications has been shown to prevent cognitive deficits, highlighting the dual roles of B cells in CNS injury (Doyle et al. 2015). Additionally, postmortem analyses of ischemic stroke patients with dementia revealed B cell infiltration (Doyle et al. 2015) and the presence of antibodies in cerebrospinal fluid that may exacerbate neural damage and cognitive decline through complement activation (Prüss et al. 2012). While the role of B cells in autoimmune diseases and ischemic stroke has been widely studied, their function in ICH remains poorly understood. Our study, using MR, identified protective associations of CD25 on IgD− CD38br, CD19 on memory B cells, and PB/PC %B cells against ICH. Conversely, it revealed causal links between subtypes such as IgD+ CD38br AC, IgD+ CD38− %B cells, IgD on IgD+ CD38dim, IgD− CD27− %lymphocytes, and transitional AC with an increased risk of ICH. These findings suggest B cells as potential therapeutic targets, opening new opportunities for innovative strategies in managing ICH (Hofmann, Clauder, and Manz 2018).

Monocytes, precursors of tissue macrophages and Dendritic cells (DCs), exhibit significant heterogeneity in their immunological functions. In humans, monocytes are primarily categorized into two subsets: the CD14highCD16− monocytes, the dominant population, and the CD16+ monocytes, which include CD14highCD16+ and CD14lowCD16+ subsets. CD16+ monocytes are known for their pro‐inflammatory properties, secreting high levels of cytokines like IL‐1β and TNF‐α in response to toll‐like receptor ligands and immune complexes (Belge et al. 2002; Ziegler‐Heitbrock 2007; Wong et al. 2011). The CD14highCD16+ monocytes, characterized by high expression of MHC class II, enhance antigen presentation, while CD14lowCD16+ monocytes, with elevated MHC class I expression, exhibit increased migratory activity but reduced phagocytic capacity. Recent studies have highlighted the role of pro‐inflammatory CD16+ monocytes in disease pathogenesis, including infectious and autoimmune diseases. In our analysis, we observed dual roles among monocyte subsets in ICH. Specifically, HLA DR expression on CD14− CD16+ monocytes was associated with protective effects, while CD64 expression on monocytes correlated with increased ICH risk. These findings emphasize the functional diversity of monocyte subsets, highlighting both their protective and detrimental roles. Targeting specific monocyte markers, such as enhancing HLA DR or inhibiting CD64, may provide novel therapeutic strategies for mitigating ICH‐related damage.

Myeloid cells, particularly neutrophils, play a major role in the inflammatory response and immune regulation, which complicates brain injury pathology (Y. Li et al. 2019). Necrotic cells in the infarcted area release pro‐inflammatory cytokines, triggering a rapid influx of immune cells. Neutrophils quickly infiltrate the brain following a stroke, where they worsen ischemic neurotoxicity by producing ROS, such as superoxide radicals and hydrogen peroxide. Additionally, neutrophils release pro‐inflammatory mediators that intensify the inflammatory response (Ruhnau et al. 2017). In a postmortem study of ICH, neutrophils and neutrophil extracellular traps (NETs) were found in the hematoma and surrounding tissues (Puy et al. 2021). In experimental ICH, neutrophils generate matrix metalloprotease and lead to vascular disruption, BBB collapse, axonal damage, and activation of astrocytic and microglial/macrophage (Moxon‐Emre and Schlichter 2011). Neutrophil depletion reduced microglia/macrophage infiltration and alleviated axonal damage and myelin fragmentation (Moxon‐Emre and Schlichter 2011). A systematic review and meta‐analysis also linked a high neutrophil‐to‐lymphocyte ratio (NLR) to poor prognosis in ICH patients (M. Shi, Li, et al. 2022). In our analysis of myeloid cell subsets, we found that CD11b on CD66b++ myeloid cell, CD33br HLA DR+ CD14dim %CD33br HLA DR+, and CD33 on CD33dim HLA DR+ CD11b− were linked to a reduced risk of ICH. Conversely, CD33dim HLA DR+ CD11b− %CD33dim HLA DR+ and HSC AC were indicative of an elevated risk of ICH. These findings suggest that different myeloid cell subsets may have distinct effects on ICH risk.

DCs are classified into two main categories: cDC and plasmacytoid dendritic cells (pDC) (Arroyo Hornero and Idoyaga 2023). Both types of cells are responsible for monitoring the local microenvironment of the immune system, which encompasses the CNS. They are highly effective in presenting antigens to T cells (C. Chen et al. 2020). Research indicates that DCs travel to the sites of cerebral injury and play a crucial role in mediating localized inflammation after experimental ischemic stroke and ICH (Ludewig et al. 2016). The reduction of brain CD11c+ cells after neuroprotective treatments in experimental stroke models implies their significant role in brain damage (Gelderblom et al. 2012). In preclinical ischemic stroke models, the suppression of myelin‐specific T cell autoimmunity decreases the number of DCs and alleviates brain damage, underscoring the critical function of antigen presentation in triggering T cell responses (Subramanian et al. 2009). Additionally, DC can initiate an antigen‐independent response; specifically, the activation of IL‐17γδ T cells by DC‐derived IL‐23 results in the influx of neutrophils into the brain affected by ischemia (Gelderblom et al. 2018). Postmortem brain tissue from patients with ischemic and hemorrhagic strokes reveals clusters of cDC, pDC, and DC–T cell interactions in both vascular and nonvascular regions (Yilmaz et al. 2009). However, the exact mechanism by which DC contribute to the poor prognosis of hemorrhagic stroke remains unclear. In our study, we found that CD62L− plasmacytoid DC and CD11c on granulocytes are associated with increased ICH risk. These results suggest that DC could be a potential target for therapeutic interventions in ICH.

In addition to the T cells, B cells, monocytes, neutrophils, and DCs that we have discussed in detail, other immune cells, including NK cells, also interact and contribute to the overall outcome of ICH. NK cells rapidly respond to acute ICH without the need for antigen priming, arriving at the hemorrhagic site earlier than other immune cells (Z. Li et al. 2020). NK cells exert cytotoxic activity and secrete chemokines and cytokines to coordinate the activity of other immune cells, thereby enhancing the local immune response (F.‐D. Shi et al. 2011). Immunostaining reveals that NK cells are more prevalent around the perihematomal region compared to other immune cell subsets, expressing activation marker CD69 and cytotoxic marker perforin (Z. Li et al. 2020). In ICH patients, brain sections show elevated counts of CD56+ or CD57+ NK cells, while circulating NK cells and their activity markers decrease. Animal model studies demonstrate that brain‐infiltrating NK cells peak within three days post‐ICH and predominantly consist of CD11b+CD27+ or CD11b+CD27− subsets (Z. Li et al. 2020). NK cell depletion leads to a reduction in brain‐infiltrating neutrophils, accompanied by a reduction in neurological deficits and smaller lesion volume. These findings suggest that NK cells play a crucial role in ICH by promoting neutrophil recruitment, thereby influencing disease progression and outcomes (Z. Li et al. 2020). Our study identified SSC‐A on HLA DR+ NK cells as being associated with a reduced risk of ICH, emphasizing the need for more research into NK cell mechanisms in ICH pathogenesis.

Despite advances in understanding the inflammatory response after cerebral hemorrhage, the neuroinflammatory process remains complex, involving interactions among neurons, glial cells, immune cells, and cytokines. While immunomodulatory therapies have shown promise in animal studies, most have failed in clinical trials, highlighting the need to better understand immune cell functions in ICH. Our study is the first to explore the causal roles of 33 immune cell subtypes in ICH, offering insights for developing targeted immunomodulatory therapies.

Using data from the GWAS Catalog and FinnGen, primarily involving individuals of European ancestry, we ensured high statistical power and reliability through robust MR analyses. However, limitations remain. Firstly, despite performing multiple sensitivity analyses, we did not fully assess potential heterogeneity and horizontal pleiotropy, possibly introducing bias. Secondly, our predominantly European ancestry sample limited the generalizability of the results. Public database sourcing of ICH cases might introduce biases related to data quality and consistency, and small sample sizes for certain immune cell subtypes in the GWAS datasets may impact reliability. Lastly, we utilized a looser threshold for evaluating results, which might increase the likelihood of false positives while more fully assessing the significant association between immune profiles and ICH. Future studies should validate these findings in diverse populations and further investigate heterogeneity and pleiotropy to ensure more accurate and applicable results.

## 5 Conclusion

This study employs MR analysis method to provide robust evidence that certain immune cell phenotypes causally contribute to the pathogenesis of ICH. These findings suggest potential avenues for future investigations and therapeutic developments.

## 6 Clinical Implications and Future Research

The identification of immune cell subtypes that influence ICH risk has significant clinical implications. Targeting these cells may herald the advent of innovative therapies to prevent and treat ICH. Future research should explore the specific mechanisms by which these immune cells affect ICH risk, including aspects such as immune cell activation, cytokine production, and vascular integrity. Furthermore, delving into the functions of both innate and adaptive immune cells is imperative for gaining a comprehensive understanding of the intricate immune landscape in ICH. Our findings provide fundamental insights into the intricate interplay between the immune cells and cerebrovascular health, laying the groundwork for personalized medicine aimed at enhancing outcomes and alleviating the burden of ICH.

## Author Contributions

**Liumei Mo**: conceptualization, data curation, formal analysis, visualization, methodology, writing–original draft, writing–review and editing, project administration. **Wei Pan**: funding acquisition, methodology, supervision, writing–review and editing, project administration. **Wenjing Cao**: writing–original draft, methodology, visualization, writing–review and editing. **Kui Wang**: conceptualization, data curation, investigation, software, formal analysis, methodology, writing–review and editing, project administration. **Li'an Huang**: funding acquisition, supervision, writing–review and editing, project administration.

## Conflicts of Interest

The authors declare no conflicts of interest.

### Peer Review

The peer review history for this article is available at https://publons.com/publon/10.1002/brb3.70263.

## Supporting information



Supporting Information

Supporting Information

Supporting Information

## Data Availability

This article and its supporting supplementary materials contain the original contributions presented in this study. Any further inquiries can be directed to the corresponding author.
